# The effect of limited access to antenatal care on pregnancy experiences and outcomes among undocumented migrant women in Europe: a systematic review

**DOI:** 10.3389/fgwh.2024.1289784

**Published:** 2024-02-06

**Authors:** Jennifer Okhianosen Osuide, Ali Davod Parsa, Ilias Mahmud, Russell Kabir

**Affiliations:** ^1^ School of Allied Health, Faculty of Health, Medicine and Social Care, Anglia Ruskin University, Essex, Chelmsford, United Kingdom; ^2^ BRAC James P Grant School of Public Health, BRAC University, Dhaka, Bangladesh; ^3^ School of Health, University of New England, Armidale, NSW, Australia

**Keywords:** antenatal care, migrant, Europe, women, undocumented migrant, health equity, maternal health

## Abstract

Women who are undocumented migrants in Europe encounter a variety of challenges while trying to access health services, including restricted access to antenatal care (ANC) despite the importance of ANC to the well-being of mothers and their infants. This study's aim was to examine the effect that limited access to antenatal care has on the pregnancy experiences and outcomes of undocumented migrant (UM) women in Europe. Systematic searches were done on PubMed, Ovid MEDLINE, Ovid EMBASE, EBSCO CINAHL Plus, and BioMed Central. From the search results, only primary research articles that reported on the pregnancy outcomes and experiences of undocumented migrants were selected. A meta-analysis was not possible because this review included information from both qualitative and quantitative studies. The data that was taken from the included publications was organised, analysed, using the Microsoft Excel programme, and then meta-synthesised. Twelve papers from seven different European nations—Belgium, France, Sweden, Denmark, Norway, Finland, and England—were included in this systematic review. Eight of the studies aimed to explore the access to and utilization of ANC by undocumented migrant women and the related pregnancy outcomes. Two of the included studies examined the pregnancy experiences of UMs and two examined the perinatal risks associated with living as a migrant with no legal status. Although heterogeneous in their specific findings most of the studies showed undocumented immigrants are more likely to experience unfavourable pregnancy outcomes and experience greater anxiety and worries due to a variety of factors than documented migrants and registered citizens. This review's conclusions demonstrate the pressing need for policy modifications and healthcare reforms in Europe to address the problems associated with undocumented migrants’ restricted access to antenatal care. It also highlights the urgent need for structural changes that will give this vulnerable population's health and well-being a higher priority. It is not just an issue of health equality but also a humanitarian obligation to address the many obstacles and difficulties undocumented migrant women endure during pregnancy.

## Introduction

The number of internally displaced people, refugees, asylum seekers, and other displaced or migrant groups is estimated to be close to 89.3 million in 2021 (1), up significantly from the estimated number of just over 68 million in 2018 (2). Additionally, 1.08 million non-European citizens were discovered to be living illegally in the European region in 2023 (3) which is up 59% of what the numbers were in 2021. This indicates that there is an annual rise in migrant numbers in the region, many of which are undocumented. Undocumented migrants (UMs) are migrants who have arrived in foreign settings, such as European nations, and who remain there without the legal authorization or documentation required by local authorities (4). Many UMs enter these nations via the regular route with a valid visa to study, work, or seek asylum; however, they later lose their status due to job loss, delays in the immigration process, and leaving an abusive partner or employer on whose status they relied. Some of these UMs have also been victims of human trafficking (5).

Universal health coverage, independent of a person's immigration status, is one of the World Health Organization's (WHO) top priorities (6). The United Nations (UN) has recognised that undocumented immigrants are among the most disadvantaged groups in society, and deserve to be protected by human rights laws. The UN has also stated that without ensuring healthcare equality for this group of people, they have a higher chance of experiencing poor treatment and discrimination (7). Primary healthcare, which addresses an important aspect of human well-being across all stages of life including sexual and reproductive healthcare (SRH), continues to present unique challenges for UMs even though countries around the world are moving towards universal health coverage where primary healthcare is essential (8). International human rights agreements binding on all European Union countries clearly establish that everyone has rights to SRH, everywhere. However, due to their frequent exclusion from accessing certain services, UMs in many of these states have limited access to necessary SHR services (9, 10). According to the Platform for International Cooperation on UMs, pregnant UMs have some limited rights to healthcare and can obtain maternity care in 21 European countries to varying degrees, ranging from delivery only to other maternal health services, including antenatal care (ANC) (5). The World Health Organisation (11) defined antenatal care (ANC) as “the care given to pregnant women and adolescent girls by qualified healthcare professionals to ensure the best health conditions for both mother and baby during pregnancy” and recommends at least eight antenatal visits during pregnancy, emphasising the need for an early start to ANC visits. Despite the significance of ANC for the health of pregnant women and the children they carry, UMs encounter many obstacles when attempting to obtain this care (12). Some of these obstacles include fear of deportation, not having legal rights to healthcare, feeling uneasy when visiting public healthcare facilities, having trouble communicating in other languages besides their native language, and being in economically dire situations (13).

Non-Governmental Organisations (NGOs) operate a variety of medical clinics to cover the gaps left by Europe's sparse availability of healthcare services for UMs. Due to a lack of or restricted access to governmental facilities, UMs may choose alternative ANC delivery strategies, such as going to these NGO clinics. According to Eick et al. (14), The standard of care provided to expectant women without documentation by NGO clinics and their role in providing basic healthcare are not without limitations. Several studies (13, 15–18) have highlighted exactly how limited, substandard, or unavailable maternal services are for UMs and what the barriers are to accessing these services in various European countries. Some studies have gone further to explore how UMs utilise the skeletal maternal services available (14, 19); and to investigate the experiences or maternal health outcomes of this target population (17, 20, 21). However, there exists a scarcity of research detailing exactly how limited access to ANC affects the pregnancy experiences of UMs living in Europe and the long-term impacts.

The existing systematic reviews have not focused on UMs specifically not asylum seekers, refugees, or legal migrants) in Europe. This review's aim is to examine the effect that this limited access to antenatal care has on the pregnancy experiences and outcomes of UMs in Europe. The objectives are: To investigate maternal health disparities between UMs and other pregnant populations in Europe. To explore the obstacles and enablers that affect (access to antenatal care for UMs in Europe. To uncover possible long-term public health implications. To inform maternal care policies and practices. Understanding and highlighting these issues has the potential to improve the access to and quality of maternal care for UMs, and to promote health equity by contributing to the evidence needed for advocacy, policy-making, policy implementation, and government-level decision-making.

## Methods

### Research question

What is the impact of restricted access to antenatal care on pregnancy outcomes and the lived experiences of undocumented migrants living in European countries?

Primary research papers that used both qualitative and quantitative study approaches were included in this review. The SPIDER search tool (Sample, Phenomenon of Interest, Design, Evaluation, Research type) was used to identify the keywords (Table 1). According to (22), the refined components of this tool are more suitable for qualitative and mixed-methods research. PRISMA, 2020 the Preferred Reporting Items for Systematic Reviews and Meta-Analyses guidelines were followed. A comprehensive search of the published literature was conducted to identify different publications. To ensure minimal bias and prevent missing pertinent studies, the literature search was conducted across many databases: PubMed, Ovid MEDLINE, Ovid EMBASE, EBSCO CINAHL Plus, and BioMed Central. The SPIDER search tool was used to find the keywords based on their suitability (for a mixed-methods systematic review).

**Table 1:** SPIDER search tool.

S- Sample	Pregnant undocumented migrants in Europe
PI- Phenomenon of interest	Limited access to antenatal services
D- Design	Published peer-reviewed literature of any research design
E- Evaluation (outcome)	Pregnancy experiences, health outcomes, Pregnancy outcomes.
Research type	Qualitative and quantitative

To get more targeted results, the Boolean operators’ conjunctions “AND” and “OR” were utilised. the databases were searched using a combination of keywords:

(Undocumented Migrants OR Undocumented Immigrants OR Illegal Immigrants OR Undocumented Women OR Migrant Women OR Migrants in Europe) AND (Antenatal care OR Perinatal care OR Access to Antenatal care OR Prenatal Care OR Maternal Care OR Utilisation of Antenatal Care) AND (Pregnancy Experience OR Pregnancy Complication OR Pregnancy Outcome OR Childbirth OR Health Outcomes).

**Figure 1:**
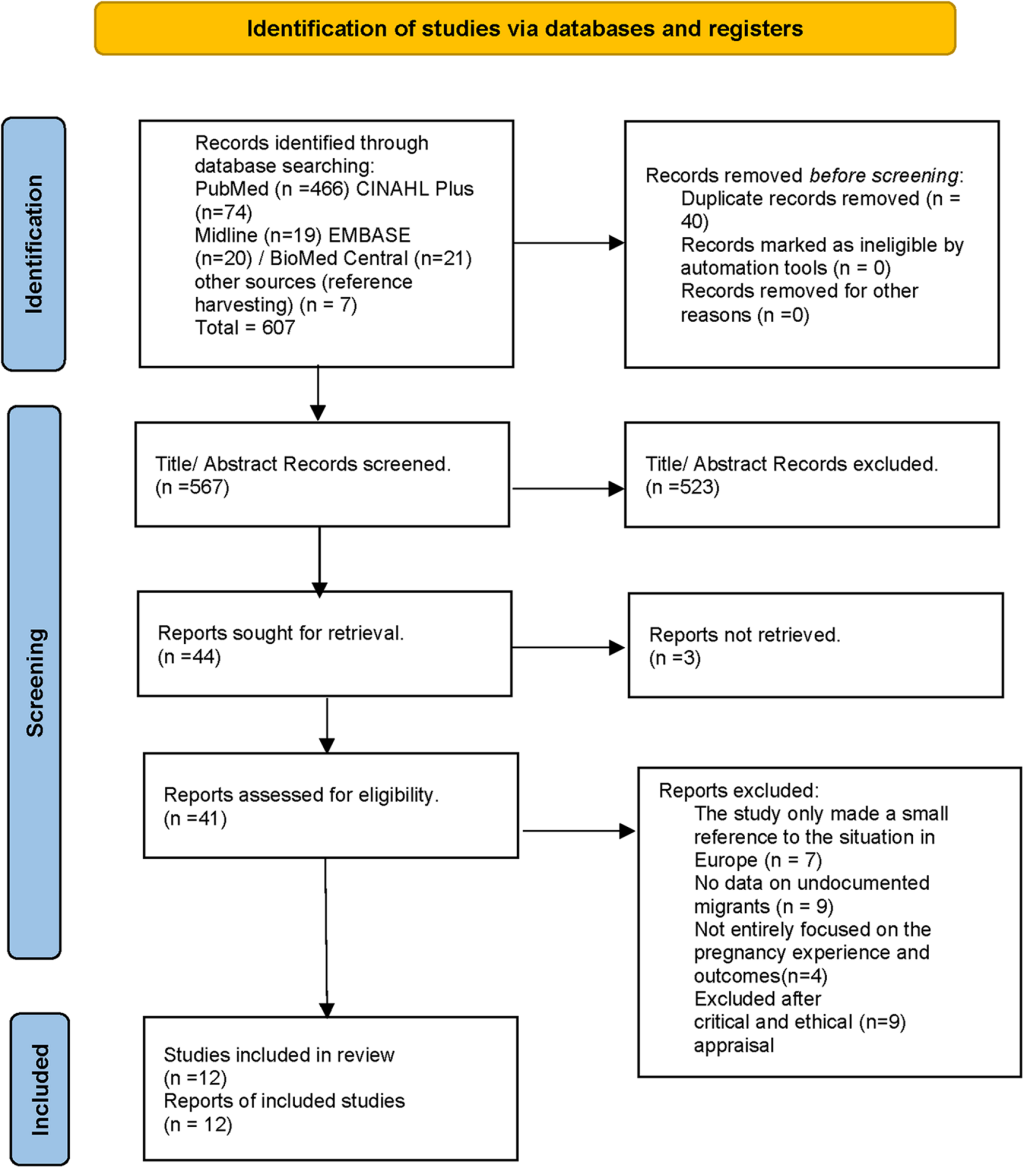
(PRISMA) 2020 flow diagram of study selection.

To narrow the scope of the search to primary, full-text, English-language, peer-reviewed papers, search limits were put in place. In order to find additional research, reference lists of relevant investigations that were found through database searching were examined (referred to as “reference harvesting”). The database search produced 607 items, seven of which were obtained by reference harvesting (see Figure 1).

The result of the literature search was 567 papers after duplicates were removed. Inclusion and exclusion criteria are presented in Table 2.

**Table 2:** Inclusion and exclusion criteria.

	Inclusion	Exclusion
S- Sample	Pregnant undocumented migrant women in Europe	Documented migrants and undocumented migrants outside Europe
PI- Phenomenon of Interest	Access to antenatal services for migrant women/undocumented migrant women.	Studies with no data regarding antenatal services for migrant women/undocumented migrant women.
D- Design	Interviews, focus groups, questionnaire, survey	case studies, interventions, intervention research
E- Evaluation (Outcome)	Pregnancy experiences, health outcomes, pregnancy outcomes.	Articles that had no data on pregnancy experiences, health outcomes, and pregnancy outcomes.
Research Type	Peer-reviewed, primary research, qualitative studies, quantitative studies, no timeframe, published in the English language.	Not peer-reviewed, languages other than English, literature other than primary research.

After applying the limits, the articles that came up in the first step of the screening process were checked for research design; systematic reviews were removed. In the second stage, the title and abstract of the studies were read through to identify the sample and study designs, these were restricted to information gathered through interviews, focus groups, questionnaires, and surveys, from both qualitative and quantitative research. Some of the papers that were scanned were omitted because they did not address antenatal care access, pregnancy experiences, or outcomes of UMs. The third stage involved screening 41 relevant research papers on Ums (S-sample), and their full-text articles were checked for information gathered about participant's experiences or outcomes regarding pregnancy or access to antenatal care. Articles that did not adequately or clearly address the PI and E criteria were not included. 12 papers were chosen for the critical appraisal stage after the inclusion and exclusion criteria were applied.

21 studies were appraised to examine the strengths and flaws of their methodology, the research validity, the reliability of findings, and the existence of biases (see Tables 3–7). The appraisal of qualitative studies was done using the CASP tool, the cohort, case-control, and prevalence studies were appraised using the JBI tool, and the Axis tool was used to appraise the cross-sectional studies. It was also done to test if the studies could provide useful answers to the questions posed by this review. This appraisal resulted in 12 studies that were included in the review.

**Table 3:** The (CASP) tool for the critical appraisal of qualitative studies.

Qualitative studies: CASP tool				Section A: Are the results valid?				Section B: What Are the results?		
Reference	Was there a clear statement of the aims of the research?	Is a qualitative methodology appropriate?	Was the research design appropriate to address the aims of the research?	Was the recruitment strategy appropriate to the aims of the research?	Was the data collected in a way that addressed the research issue?	Has the relationship between researcher and participants been adequately considered?	Have ethical issues been taken into consideration?	Was the data analysis sufficiently rigorous?	Is there a clear statement of findings?	How valuable is the research?
Hargreaves et al. (10)	+	+	+	+	+	+	+/-	+	+	+
Lyberg et al. 2012	+	+	+	+/−	+	−	+	+/−	+	+/−
Funge et al. (13)	+	+	+	+	+	+	+	+	+	+
Barkensjö et al. (16)	+	+	+	+/−	+	+	+	+	+	+
Nellums et al. (17)	+	+	+	+	+	+	+	+	+	+
Kvamme and Ytrehus, (18)	+	+	+	+	+	+	+	+	+	+
Castaner et al. 2022	+	+	+	+	+	+	+	+/−	+	+

(+) = item adequately addressed, (−) = item not adequately addressed, (+/−) = item partially addressed.

**Table 4:** The JBI tool for the critical appraisal of prevalence studies.

Reference	Was the sample frame appropriate to address the target population?	Were study participants sampled in an appropriate way	Was the sample size adequate	Were the study subjects and the setting described in detail	Was the data analysis conducted with sufficient coverage of the identified sample	Were valid methods used for the identification of the condition	Was the condition measured in a standard, reliable way for all participants	Was there an appropriate statistical analysis?	Was the response rate adequate, and if not, was the low response rate managed appropriately
Wendland et al. (19)	+	+	+	+	+/−	+	+	+	+/−

(+) = item adequately addressed, (−) = item not adequately addressed, (+/−) = item partially addressed.

**Table 5:** The JBI tool for the critical appraisal of case-control studies.

Reference	Were the groups comparable other than the presence of disease in cases or the absence of disease in controls?	were cases and controls matched appropriately?	Were the same criteria used for identification of cases and controls?	Was exposure measured in a standard, valid and reliable way?	Was exposure measured in the same way for cases and controls?	Were confounding factors identified?	Were strategies to deal with confounding factors stated?	Were outcomes assessed in a standard, valid and reliable way for cases and controls?	Was the exposure period of interest long enough to be meaningful?	Was appropriate statistical analysis used?
Faurholdt et al. (20)	+	+	+	+	+	+	+/−	+	+	+

**Table 6:** The JBI tool for the critical appraisal of cohort studies.

Reference	Were the two groups similar and recruited from the same population?	Were the exposures measured similarly to assign people to both exposed and unexposed groups?	Was the exposure measured in a valid and reliable way?	Were confounding factors identified?	Were strategies to deal with confounding factors stated?	Were the groups/participants free of the outcome at the start of the study (or at the moment of exposure)?	Were the outcomes measured in a valid and reliable way?	Was the follow up time reported and sufficient to be long enough for outcomes to occur?
Eick et al. (14)	NA	NA	+	+	+/−	+	+/−	+
Tasa et al. (23)	+	+	+	+	+/−	+	+	+
Eslier et al. (21)	+	+	+	+	+	+	+	+/−
Liu et al. (24)	+	+	+	+	+	+	+/−	NA
Schoenborn, Spiegelaere,and Racape, (25)	+	+	+/−	+	−	+	+	+
Boerleider et al. 2015	+	+	−	+/−	−	+	+/−	−
Eslier et al. (26)	+	+	+	+	+	+	+	+/−

**Table:** 

Reference	Was follow-up complete, and if not, were the reasons to loss of follow-up described and explored?	Were strategies to address incomplete follow-up utilized?	Was appropriate statistical analysis used?
Eick et al. (14)	−	+/−	+
Tasa et al. (23)	+	+	+
Eslier et al. (21)	+	+/−	+
Liu et al. (24)	NA	NA	+
Schoenborn, Spiegelaere,and Racape, (25)	NA	NA	+
Boerleider et al. 2015	−	−	+
Eslier et al. (26)	+/−	+/−	+

(+) = item adequately addressed, (−) = item not adequately addressed, (+/−) = item partially addressed, NA = Not Applicable.

**Table 7:** The AXIS tool for the critical appraisal of cross-sectional studies.

Reference	Introduction						Methods			
	Were the aims/ objectives of the study clear?	Was the study design appropriate for the stated aim(s)?	Was the sample size justified?	Was the target/ reference population clearly defined? (is it clear who the research was about?)	Was the sample frame taken from an appropriate population base so that it closely represented the target/ reference population under investigation?	Was the selection process likely to select subjects/ participants that were representative of the target/ reference population under investigation?	Were measures undertaken to address and categorize -non-responders?	Were the risk factor and outcome variables measured appropriate to the aims of the study?	Were the risk factor and outcome variables measured correctly using instruments/ measurements that had been trialed, piloted or published previously?	Is it clear what was used to determine statistical significance and/or precision estimates? (e.g.,*p*-values, confidence intervals)
Doetsch et al. (15)	+	+	+	+	+	+	+	+	+	+
Almeida et al. 2014	+	+	+	+	+	+	+	+	+	+
Ankert et al. 2021	+	+	−	+	+/−	+/−	−	+	+	+
Lelong et al. 2015	+	+	+/−	+	+	+	−	+	+	+
Wolff et al. 2004	+	+	+	+	+	+	+/−	+	+/−	+

**Table:** 

Reference	Results						Discussion		Others	
	Were the methods (including statistical methods) sufficiently described to enable them to be repeated?	Were the basic data adequately described?	Does the response rate raise concerns about non-response bias?	If appropriate, was information about non-responders described?	Were the results internally consistent?	Were the results presented for all the analyses described in the methods?	Were the authors’ discussions and conclusions justified by the results?	Were the limitations of the study discussed?	Were There Any funding sources or conflicts of interest that may affect the authors’ interpretation of the results?	Was ethical approval or consent of participants attained?
Doetsch et al. (15)	+	+	+/−	+	+	+	+	+	+	+
Almeida et al. 2014	+	+	−	+	+	+	+	+	+	+
Ankert et al. 2021	+	+	+/−	−	+	+	+	−	+	+
Lelong et al. 2015	+	+	+/−	−	+	+	+	+	−	+
Wolff et al. 2004	+	+	−	NA	+	+	+	+	+	NS

(+) = item adequately addressed, (−) = item not adequately addressed, (+/−) = item partially addressed, NS = Not Stated or “I do not know”, NA = Not Applicable.

The Microsoft Excel programme was used to extract study data. The data extracted included the in-text citation of the article; the study setting; the study design; the sample size; the aim of the study; the study findings and the limitations of the study. This systematic review contains data from both qualitative and quantitative research, and therefore, a meta-analysis could not be conducted. After the extraction and organization of data from the included articles, a meta-synthesis was carried out.

## Results

This systemic review included 12 studies from seven different countries; these studies were conducted in the European countries of Belgium, France, Sweden, Denmark, Norway, Finland, and England. Eight of the studies aimed to explore the access to and utilization of ANC by UM women and the related pregnancy outcomes. Two of the studies explored the pregnancy experiences of UMs and two examined the perinatal risks associated with living as a migrant with no legal status. Included were four qualitative studies that used in-depth interviews to gather their data. A self-administered questionnaire and information from birth and death certificates gleaned from national population registers were used to collect data for six cohort studies. Quantitative data from a case-control study and a prevalence study were also included in the analysis. The studies included various samples differing in immigration status, social positions, and education levels. Some Characteristics, designs, and summaries of the findings are presented in Table 8.

**Table 8:** Data extraction table (characteristics of the 15 papers included in the review and summary of their findings).

Reference	Study setting	Study design, sample size	Aim	Study findings	Limitations
Eslier et al. (21)	Data was collected from four maternity units around Paris between 2010 and 2012	A Prospective Cohort Study including a total of 9,599 women.	The study aim was to evaluate the relationship between the migrant profile of women, considering both their legal status and place of birth, and adverse maternal outcomes.	Undocumented migrants had been in France for a shorter period of time and encountered barriers to accessing healthcare services. Compared to French-born mothers, they exhibited a greater incidence of severe maternal morbidity.	Due to incomplete questionnaires, missing pregnancy outcome data, women giving birth abroad, or women who were lost to follow-up, a sizable number of women were removed from the study.
Schoenborn, Spiegelaere,and Racape, (25)	From the Belgian civil registration system, data was retrieved concerning all singleton babies born between 1st January 2010 and 31st December 2016, without links to their mothers’ NPR registration status.	A Cohort Study including Documented women *n* = 854,689 Undocumented women *n* = 16,594	To examine the relationships between legal status and pregnancy outcomes based on nationality and socioeconomic position.	Nearly 2% of deliveries throughout the research period were to mothers who were not registered in the NPR. NPR registration plays an important role in perinatal health. The higher perinatal mortality for non-European UMs may be partially explained by their dire social and economic circumstances.	Given that the unique identifier pertained to births and not mothers, the study was unable to link individual births to the same mother over the course of the seven-year period.
Liu et al. (24)	Women without a permanent personal identity number receive perinatal treatment using a temporary number. In Sweden, 46 delivery hospitals with individual catchment areas and neighbourhood prenatal facilities account for around 99% of all births.	Register-based Cohort study including Swedish women *n* = 254,973, Undocumented women *n* = 31,897.	To investigate the perinatal health outcomes of female refugees between 2014 and 2017, with a particular emphasis on immigration status as a factor affecting prenatal health among migrants.	Prior to getting pregnant, women who were undocumented immigrants and asylum seekers had lower self-rated health than refugee women but received less ANC. They also had a higher risk of premature birth than refugee women.	The results might not be directly extrapolated to different contexts and environments.
Nellums et al. (17)	Doctors of the World (DOTW), a global human rights NGO, works to make it easier for excluded or marginalised people to obtain healthcare. From June to December 2017, the study was carried in their clinic in London.	A Qualitative study using semi-structured interviews. The total sample of undocumented women was *n* = 20.	To examine the experiences of pregnant UMs in England, as well as the factors that affect their access to care and their subsequent health outcomes.	Restricted agency, interconnected difficulties, and a recurring cycle of uncertainty characterised what women felt they experienced with maternity care.	The study used a variety of interpreters who had different approaches.
Faurholdt et al. (20)	Nationwide registers from Statistics Denmark and hospital journals from the seven largest hospital wards in Denmark from 1 January 2011 to 31 December 2018.	Retrospective Case-Control Study. A total of 882 undocumented pregnant migrant women and 3,528 matched controls.	To investigate the links between pregnant migrant women without legal status and the chance of a stillbirth or early birth.	According to the study, compared to the control group, pregnant migrant women without legal status had a risk of stillbirth that was more than three times greater and a risk of preterm delivery that was higher.	The study was unable to compare the findings of the sub-analysis with the body of previous research since there was no standard approach for identifying a foundation of residency and because the categories described in the study were developed from what made sense in a Danish setting.
Barkensjö et al. (16)	The study included immigrants without documentation and EU nationals without a valid residency visa who were or had recently given birth in Sweden. Multiple individuals were involved in recruitment, including two nurses, two authors, a midwife at the hospital, and a cultural assistant from an NGO.	Qualitative study using interviews. Thirteen women from ten different countries were interviewed.	To give a comprehensive account of how women who were undocumented migrants in Sweden experienced clinical interactions throughout pregnancy and during labour.	The study found that negative experiences caused anxiety and psychological distress. Positive interactions, on the other hand, encouraged confidence in medical professionals, self-reliance, and satisfaction.	It could be claimed that the ladies who took part in this study may have been prevented from expressing themselves freely because eight of the women spoke through interpreters. Participants admitted they feared that if they voiced criticism, they might be denied care.
Funge et al. (13)	The study was done with the help of a clinic run by a non-governmental organization (NGO) in Denmark.	A qualitative study including 21 undocumented migrant women in Denmark.	To investigate the maternal experiences and access to maternity care services for UMs in Denmark throughout pregnancy.	The study revealed that undocumented migrants faced several barriers to accessing healthcare and thus relied on alternative strategies such as attending NGO clinics and connecting with informal networks.	The data gathered did not contain information on lifestyle, general health, including mental health, or migratory history. The transferability of the study is impacted by the fact that undocumented migrants's rights are not the same in every developed country.
Wendland et al. (19)	For pregnant women who visited three clinics that specialise in providing treatment for undocumented migrants between August 2011 and August 2014, the study collected individual-level data on the frequency and outcomes of tests for sexually transmitted diseases	A prevalence study. Pregnancy-related episodes *n* = 329.	To compare the prevalence of infection between undocumented and documented migrants and to determine the frequency of screening among undocumented migrants.	The study demonstrated that undocumented migrants had a higher frequency of HBV than documented migrants and that they had a lower chance of getting tested for HIV, HBV, and syphilis.	The study was unable to determine if the women who showed up at a clinic were a good representation of the pregnant undocumented migrants in Denmark.
Eick et al. (14)	Oslo and Bergen, Norway's capital and second-largest city, respectively, were the study's locations. Records were gathered from the two non-profit clinics that were formed as a result of a partnership between Church City Mission and the Norwegian Red Cross to fill the gaps in illegal immigrants’ access to primary care.	A cohort study of 500 women during the study period.	To investigate the use of NGO clinics by pregnant undocumented migrant women and how this impacts their maternal health. Additionally, the study sought to investigate if unfavourable maternal and perinatal outcomes were significantly related to the region of origin.	Based on the origin of the women, the study found significant variation in their health-seeking behaviour, and also discovered a low risk (6%) of recognised comorbidities and unfavourable gestational conditions. The women did, however, face a high risk of induced abortions, poor maternal and neonatal outcomes, and sexually transmitted diseases.	Due to the fact that some of the women did not visit the NGO clinics for assistance with their additional pregnancies, these pregnancies were not included in the study.
Kvamme and Ytrehus, (18)	The Health Centre for Undocumented Immigrants in Oslo served as the source for the recruitment of study participants.	Qualitative study using interviews. 8 undocumented migrant women and 8 healthcare personnel.	To examine the subjective experiences of undocumented migrant women with regard to their health issues and access to healthcare.	Both the women and medical professionals discussed the difficulties in obtaining the essential medical care from the Norwegian health care services as well as the lack of access to these services. They connected the social circumstances of the women to their health issues.	A health facility for undocumented migrants was where participants were found. In this study, there was no representation of the populations who may be most disadvantaged and who do not interact with this kind of health centre.
Eslier et al. (26)	Four maternity units around Paris between 2010 and 2012	A cohort study that included 9,599 women	To determine if there is a relationship between a woman's legal status and her insufficient prenatal care utilisation (PCU) in France, a country where healthcare is meant to be accessible to everyone.	Migrant women are at a higher risk of underutilising ANC and a higher risk of prenatal complications compared with women born in France.	The significant number of women who were excluded due to incomplete questionnaires, missing data on pregnancy outcomes, or lost to follow-up because they gave birth elsewhere remains a limitation.
Tasa et al. (23)	UMs have had access to maternal health care services in Helsinki. This study was conducted Finland.	A cohort study with a population of all 60 UMs.	To examine the utilisation of health services and the maternal health outcomes of undocumented women in Helsinki, Finland's capital, and to compare the findings with those of all Finnish expectant mothers.	The findings of this study highlight three crucial issues: undocumented women frequently sought out maternal health care services late in pregnancy, insufficient prenatal care was provided, and undocumented women had a higher prevalence of infectious diseases.	The study's main limitation is its very small study sample, and it is impossible to rule out the potential that some undocumented women were overlooked.

### Barriers to accessing antenatal care

One of the most frequently occurring situations recorded in several of the studies was the unavailability or under-utilization of ANC services by UMs. In some cases, women presented late for their first ANC visit, some women had well below the recommended number of visits and some did not utilise the ANC services provided by public health centers or NGO clinics at all. Most of the studies pinned this phenomenon on certain interconnected factors.

### Knowledge gaps

Due to their legal situation and the national rules of the country they resided in, some studies revealed that UMs felt they had little control over their lives and bodies and were unaware of the resources that were available to them or how to get the help they needed (14, 17, 18). A study by Barkensjo et al. (16) uncovered a lack of understanding of how to interact with UMs among healthcare workers in Sweden. These medical personnel would frequently inquire about UMs’ rights to healthcare services, and they were frequently uninformed of the barriers individuals would face in doing so, such as even merely picking up prescription drugs from the pharmacy. Although they were legally entitled to some levels of care, many of the women in this study recalled instances in which they were denied access to antenatal care, specialist treatment, and emergency care. The state medical aid (AME) system was established in France in 1999 and allows UMs to claim free care; however, some UMs are unaware of these provisions, and some medical professionals turn away women who have no AME coverage (26).

### Fear of deportation

Funge et al. (13) identified UMs’ fear of being deported to their home countries as one of the primary barriers preventing them from accessing and using ANC in Denmark. All of the study's participants eventually went to public hospitals, but only when they felt it was absolutely necessary, and for some only when they believed that the only alternative was “hospital or death.” Another study suggested that the biggest deterrent for UMs from seeking medical attention in Norway was their fear of being discovered by the police or immigration officers (18). One participant in this study noted that the only reason she went to the health centre was that she felt she was going to die. The women in this study were unaware of the Norwegian healthcare providers’ need to protect patient confidentiality and were still unsure of whether they would be discovered by the authorities if they sought medical care. For undocumented women, pregnancy presents a particularly dangerous period because it makes them more visible and vulnerable. These women were hesitant to seek medical attention and were unsure of what to do.

### Socioeconomic conditions

Due to their restriction from social assistance and legal employment, numerous studies have discovered that UMs’ lives are typically ruled by poverty and instability (25). These investigations also revealed that UMs did not have easy access to affordable accommodation and that there were few job opportunities available, most of which were on the black market, where wages were low and working conditions were poor. Despite numerous requests from health professionals for social equity and reductions in health disparities, healthcare services in the UK, for instance, continue to be fee-based for those with undocumented immigration status (27). The issue remains: How do UK UMs pay for this level of care if they are unable to legally work and support themselves? Frequent, specialised ANC is necessary due to underlying medical issues which go undetected or undertreated; yet, these women may delay or be prevented from receiving care due to the possibility of paying exorbitant fees and their fear of immigration penalties, including deportation (28). Several studies noted that social isolation and poverty were sources of physical and mental stress for UMs, pointing out that the women who had paid jobs had regular routines in their daily lives. They could build bonds and relationships with friends, go to work during the day, and earn enough to afford rest and privacy (18).

### Language and culture

Language barriers that may make it difficult for UMs to properly comprehend medical instructions and communicate with healthcare professionals may increase the difficulty with which UMs access prenatal care (24), as a result, they may be less enthusiastic or motivated to use ANC services. Henry, Beruf, and Fischer (29) found that in addition to the women's language barriers, hospitals’ inability to offer interpreters also contributed to their worry about giving birth and their feelings of helplessness when treatment was administered against their will. When techniques or treatments could not be described, it also resulted in detrimental health effects, such as infections or difficulties breastfeeding. Even when they are able to obtain ANC to some extent, UMs rarely use it due to cultural and religious differences (30). According to some studies, the absence of documentation could impede UMs’ ability to communicate with the healthcare system. The language and cultural obstacles that already afflict migrants generally are exacerbated by this status (26). Women in the Barkensjo et al. (16) study interviews expressed shock and gratitude to the medical staff members who provided them with the assistance they needed, such as by providing an interpreter or directing women to specialised care for physical/psychological examination. They valued the presence of midwives who spoke their local language.

### Impact on pregnancy outcomes

#### Maternal outcomes

Eslier et al. (21) found that undocumented migrants in France had a higher risk of severe maternal morbidity (33/715 [4.6%] vs. 129/4523 [2.9%]; absolute difference 1.7%, 95% CI 0.4%–3.6%; a OR 1.68, 95% CI 1.12–2.53), when compared to the reference group, it was discovered that undocumented migrants had hypertensive problems at least twice as prevalent as non-migrants or legal migrants. However, this study indicated that only undocumented migrants born in sub-Saharan Africa were at a significantly increased risk when considering both their location of birth and their legal status. Schoenborn, Spiegelaere, and Racape (25), found that the odds ratios for perinatal mortality in Belgium were statistically significantly higher for UMs compared to women with a nationality from Belgium who were captured on the National Population Register (NPR). The nationality groups of UMs with the strongest ORs were women from EU15 countries (OR (95% CI) 7.3 (6.0–8.95), *p* < 0.0001), followed by Belgium women without an NPR number (OR (95% CI) 4.3(3.3–5.4), *p* < 0.0001). Lui et al. (24) uncovered that UM in Sweden have a higher risk of poor self-rated health with an adjusted risk ratio (RR) of 1.84% and 95% confidence interval (95% CI) of 1.72–1.97, and were more likely to have Caesarean sections than refugee women or their Swedish counterparts. They were also more likely to have missed postpartum care visits (RR 1.15, 95% CI 1.10–1.22) compared to Swedish women but less likely to have severe postpartum haemorrhage (RR 0.78, 95% CI 0.62–0.98 and RD −12.7, 95% CI −23.2 to −2.2 per 1,000 births).

Tasa et al. (23) found that the prevalence of HIV (*p*-value <0.001) and HBV (*p*-value =0.007) was significantly higher amongst UMs in Finland compared with all other groups of pregnant women with no statistically significant difference between the proportion of vacuum-assisted deliveries or caesarian sections amongst undocumented women and all pregnant women. A similar study from Denmark showed that the prevalence of HBV was higher in UM than in DM (SPR: 2.4; 95% CI: 1.1–5.3). The SPR of 2 (95% CI: 0.5–8.0) for HIV was not statistically significant, potentially due to the small sample size of UMs (19). One study from Norway reported a high frequency of induced abortions and emergency caesarean sections among an UMs population that attended a free NGO clinic (from their first antenatal care visit at the clinic to the end of pregnancy) however, there was no reference group in this study (14).

#### Infant outcomes

One study from Denmark found that UMs were at a higher risk of stillbirth and preterm birth than the control group. A higher adjusted odd of experiencing stillbirth (aOR 3.50; 95% CI 1.31–9.38) and preterm birth (aOR 1.41; 95% CI 1.04–1.93) were observed among the undocumented pregnant migrant women compared with the control group (20) whereas (23), in their study uncovered no preterm deliveries (<37.0), low birth weight babies (<2,500 g), nor stillbirths. The rates were higher for preterm birth (RR 1.47, 95% CI 1.21–1.79 and RD 19.3, 95% CI 7.6–13.0 per 1,000 births), and low birth weight (RR 1.36, 95% CI 1.11–1.66 and RD 15.9, 95% CI 3.9–28.0 per 1,000 births) among UMs in Sweden compared to their Swedish counterparts (24). One Belgian study reported that infants born to UMs with a nationality from outside of the European region were significantly more at risk of perinatal mortality, compared to infants born to registered immigrants (pooled OR (CI 95%): 1.5 (1.1–2.1), *p* = 0.02) (25). The same study also observed a significant excess of prematurity among unregistered mothers in all nationality groups except for Turkish and South American mothers.

### Pregnancy experiences of undocumented migrant women

#### Emotional distress and stressors

According to research by Barkensjo et al. (16), fear among UMs in Sweden developed as a result of potential medical errors made by untrained healthcare workers. This fear was mainly fueled by worries that they might be discovered during visits to the doctor and deported as a result. Several women in this study received invoices after their kids were born because they had been misinformed that they would not be charged for their trips to the prenatal care centre. These encounters caused the women to feel extremely distressed, anxious, and apprehensive, which made them start to fear for their personal safety as well as the lives of their unborn children. Many of them felt they had nowhere to turn with their complaints, which exacerbated these feelings.

According to another study, UMs in Denmark who relied on NGO clinics reported having serious worries about experiencing a medical emergency—such as bleeding or giving birth—after clinic hours. When questioned about it, the women gave a helpless response in which they just conveyed their grief. Uncertainties regarding labour and delivery care had an impact on this study's female participants. Many expressed uncertainties about where to go and anguish about issues including whether they would be permitted to get treatment during childbirth and whether the lack of documentation would compromise the standard of care (13). Due to their immigration status, four of the women who took part in a Norwegian study temporarily resided with acquaintances. While the family members they lived with were at work, some others had to be gone all day. They found it unpleasant and humiliating since they felt over-dependent on others and like they were a heavy burden. Even their daily meals were another source of stress and anxiety, having to wonder about where to live and what to eat. When the interviews for this study took place, only three of the women were employed in domestic work in the black market. Only three of the women were working as domestic helpers on the black market when the interviews for this study were conducted. They made about 60 Norwegian kroner an hour, which is relatively little. One of the women said that she did not eat a lot because she had to pay for her phone and public transport (18). Similar research from England reported that UMs faced a variety of stressors that were complicatedly shaped by the interconnection of their immigration, financial, and health situations. Participants in this study indicated how financial issues, such as having trouble paying for housing, food, transportation, and medication, intersected with the pressures related to their pregnancy. These women were charged for services but were not permitted to work legally because they lacked proper documentation (17).

#### Perceptions of discrimination

In some instances, how undocumented UMs are treated by medical professionals depends on their socioeconomic and legal status. Some studies reported that UMs felt misunderstood and interrogated in healthcare settings. The way the women were treated was said to have given rise to worries that medical staff would treat them poorly or that they would be disregarded. Women often felt discriminated against when their needs and demands were not accommodated, such as when they were not allowed to see a female physician despite having strong religious convictions. Sometimes, healthcare workers differentiated between UMs and women of Swedish descent, the women said they felt undervalued at these times and that they had not received equal treatment. One woman gave the following account of her experience: “I had a catheter and was in a lot of pain. I could barely reach out for my pain medication. When I said that I was hungry no one came with food. I barely had the energy to feed the baby.” (16).

One participant in a study by Nellums et al. (17) recounting her experience said “One midwife…she was rude to me, said, ‘Hey, why don't you go back where you came from?’… I started crying because it was hurting me, tears came out of my eyes, and I said ‘I can't go back, I'm so sorry for that, I can't…”. In a Danish study, UMs felt that the restricted access to maternity care was unfair. The participants had a strong impression of their right to care due to their pregnancy and real concern for the welfare of their unborn baby, with one of them saying: “When I don't have papers. I feel sad about that. Why should my child not get the same help as other children? A child is a child” (13). Participants in this study agreed that access to and the standard of care would increase if a staff member liked a woman from a certain country or showed sympathy for the woman's condition. Access to care was therefore considered as being determined more by the individual healthcare professional than by laws and legal rights. Psychosocial stress triggered by socioeconomic conditions, feelings of being alone, prejudice, and anxiety (31) as a result of constantly having to live in the shadows, can negatively affect the overall health of mothers and their unborn babies especially as pregnancy distress has been associated with issues like spontaneous preterm birth (32, 33).

## Discussion

This systematic review of literature from seven countries in Europe reveals a connection between limited access to ANC (maternal health inequalities) and adverse maternal experiences and outcomes for UMs, despite the diversity of study data and findings. Although heterogeneous in their specific findings most of the studies demonstrate that UMs experience greater anxiety and worries due to a variety of factors than documented migrants and registered citizens and have greater chances of adverse pregnancy outcomes. These factors such as language and cultural differences, socio-economic conditions, and fear of deportation also double as some of the barriers to accessing ANC. Eslier et al. (21) hypothesised that prenatal care utilisation was inadequate for UMs, both in terms of quantity and quality, and suggested that socioeconomic and language barriers may be to blame for this group of women's underutilisation of ANC services in comparison to non-migrants and other categories of migrants. In spite of the fact that ANC is essential for the detection, monitoring, and treatment of several pregnancy-related health problems, UMs still find it difficult to access their legal rights, making it difficult for them to access and receive care. According to Schoenborn, Spiegelaere, and Racape (25), UMs make use of family planning and contraception less frequently and are therefore more likely to become pregnant unintentionally, which has been associated to worse maternal and infant health outcomes.

Eslier et al. (26) discovered that the prevalence of pregnancy complications among undocumented migrants was at least twice as high as that of non-migrants or legal migrants, and Vanneste et al. (34) found that UMs who did not have Urgent Medical Care (AMU) coverage were more likely to give birth prematurely and to have babies with lower birthweights than women who social security covering, whether through the French AMU or public health insurance. These arguments add credence to the idea that inadequate prenatal care contributes to these health disparities. Although many European countries only offer certain health care services for UMs, effectively disregarding human rights laws with serious consequences, Sweden offers equal rights to maternity care. This is however insufficient to ensure equal access to care for UMs as there is still a low reported maternity care usage and a higher risk of adverse pregnancy outcomes (24), equal rights do not translate to equal access if authorities do not develop and enforce the right regulations. Similarly, UMs in Finland have been able to access public health care since the year 2013 but Tasa et al. (23) found that they began prenatal care on average 10 weeks later than all Finnish pregnant women did. This goes to show not only that Inadequate prenatal care is given to UMs but that UMs themselves delay seeking this care. In Finland in 2018, pregnant women without documentation received eight fewer prenatal appointments on average than other pregnant women, and some did not receive any prenatal treatment at all. There is a lack of information and knowledge among health professionals and UMs about their rights and levels of healthcare entitlements available to them. Authorities consistently fail to notify UMs of their rights, and research has shown that this is a significant barrier to migrant patients receiving good healthcare. Both consumers and providers of healthcare should be aware of these entitlements (18).

Studies from Sweden and Denmark have emphasised the value of a trusting doctor-patient relationship in prenatal care for UMs, and UMs have expressed a sense of security and welcome when visiting NGO clinics. UMs pointed out once more how crucial it is for NGOs, religious groups, and cultural doulas to be involved in supporting them and catering to their healthcare needs. Women appeared to only seek perinatal care in the later stages of pregnancy due to a lack of understanding regarding their healthcare rights, and NGOs were said to have played a vital role in educating these women about their rights. This support was especially crucial for those who had previously encountered healthcare professionals and had a bad experience (16). These studies also showed that UMs who use NGO clinics frequently receive subpar ANC and are at a high risk of having negative pregnancy outcomes. even though research has demonstrated the healthcare gaps these clinics fill in Europe (13). Due to structural vulnerabilities on the one hand and inadequate volunteer resources on the other, NGO clinics find it difficult to provide full medical care services for marginalised populations (14). Being on the move frequently and depending on the goodwill of others is particularly humiliating for UMs according to their accounts and based on suggestions and encouragement from their networks in their home countries, some of the women had self-medicated instead of presenting at even the NGO clinics. One of the women claimed that she got the medicine from friends or had it shipped from home to deal with her health issues (18). Sometimes UMs will turn to friends or the internet for health-related advice, but these are not always trustworthy sources and should generally only be heeded with caution (13).

These studies have emphasized how marginalized and vulnerable UMs are (35) and the need to improve their socio-economic situations and access to health services thereby taking a huge step towards tearing down the menace of health inequalities. The continued late or null presentation of UMs to ANC may result in the delayed detection of pregnancy complications and infectious diseases which may be dangerous or even fatal for the mother and unborn child. Maternal and child morbidity and mortality and the spread of undetected infectious diseases like HIV and HBV should be public health priorities in need of immediate action regardless of the legal status of the population of interest.

### Strengths and limitations

There are many potential limitations of this review, firstly a literature search was conducted using major electronic databases and no other databases were searched and the grey literature databases were also not part of the search. Secondly, articles with full available texts and published in English languages were only included. Despite having some limitations, the findings of the review could be generalized to women of childbearing age from Europe and can be useful for policymakers to improve access to antenatal care services.

## Conclusion & recommendations

The findings of this review emphasize the urgent need for policy modifications and healthcare reforms in Europe to address the problems associated with undocumented migrants’ restricted access to antenatal care. Governments need to amend, develop, and implement policies to guarantee that every woman, undocumented or not, has access to basic healthcare services without any repercussions. Policies need to be implemented to alleviate the fear of deportation among pregnant UMs, ensuring that accessing healthcare does not put their legal status or lives at risk. Improving access and empowering people to utilise services requires removing administrative and legal obstacles. To properly serve the diverse community of UMs, healthcare professionals need to receive training in healthcare policy, cultural competency, and language proficiency. Communication between midwives, society, and UMs can be facilitated by medically qualified interpreters and personnel who understand and can stand as cultural mediators. This will ensure that healthcare providers have and are able to deliver the right information in the most appropriate and clear manner. Lack of or inaccurate information is one of the major sources of emotional stress and anxiety for UMs, recognizing the emotional distress faced by undocumented migrant women, mental health support should be integrated into antenatal care services. Community organizations and NGOs play a vital role in supporting undocumented migrant women. Investment in these organizations can help bridge the gap in healthcare access. This review highlights the urgent need for structural changes that will give this vulnerable population's health and well-being a higher priority. It is not just an issue of health equality but also a humanitarian obligation to address the many obstacles and difficulties undocumented migrant women endure during and after pregnancy. Lessons learned from this review should guide the development of antenatal care policies and practices that will ultimately improve the health outcomes and experiences of undocumented immigrant women in Europe. Further research needs to be done to uncover ways of improving the current precarious situation of UMs and their access to maternal healthcare through policy change and aggressive healthcare and health policy education interventions.

## Data Availability

The original contributions presented in the study are included in the article/Supplementary Material, further inquiries can be directed to the corresponding author.
